# FoxO1 controls lysosomal acid lipase in adipocytes: implication of lipophagy during nutrient restriction and metformin treatment

**DOI:** 10.1038/cddis.2013.404

**Published:** 2013-10-17

**Authors:** D Lettieri Barbato, G Tatulli, K Aquilano, M R Ciriolo

**Affiliations:** 1 Department of Biology, University of Rome Tor Vergata, Via della Ricerca Scientifica, Rome 00133, Italy; 2 Università Telematica di Roma San Raffaele, Via di Val Cannuta, Rome 00166, Italy; 3 IRCCS San Raffaele, Biochemistry of Ageing, Via di Val Cannuta, Rome 00166, Italy

**Keywords:** aging, ATGL, lipid metabolism, adipose tissue, autophagy

## Abstract

Finding new molecular pathways and strategies modulating lipolysis in adipocytes is an attractive goal of the current research. Indeed, it is becoming clear that several human age-related pathologies are caused by adipose tissue expansion and altered lipid metabolism. In the present work, we show that transcription factor forkhead homeobox type protein O1 (FoxO1) is upregulated by nutrient restriction (NR) in adipocytes and exerts the transcriptional control of lipid catabolism via the induction of lysosomal acid lipase (Lipa). An increased autophagy and colocalization of lipid droplets (LDs) with lysosomes was observed implying lipophagy in Lipa-mediated LDs degradation. Interestingly, we found that metformin (Metf), a biguanide drug commonly used to treat type-2 diabetes, exerts effects comparable to that of NR. Actually, it was able to elicit FoxO1-dependent Lipa induction as well as LDs degradation through lipophagy. Moreover, we demonstrate that, during NR or Metf treatment, free fatty acids released by Lipa are directed toward AMP-activated protein kinase-mediated mitochondrial oxidation, thus maintaining energetic homeostasis in adipocytes. In conclusion, our data show that lysosomal-mediated lipid catabolism is activated by NR in adipocytes and give further support to the use of Metf as a NR mimetic to combat age-related diseases associated with altered lipid metabolism.

Biological aging is typically characterized by a progressive increase in body fat mass. Excess or abnormal fat accumulation may set adverse effects on health and decrease life expectancy.^1^ Actually, heightened adipose tissue (AT) accumulation, especially of visceral AT, amplifies the risk of developing various age-related diseases, including cardiovascular disease, type-2 diabetes mellitus and certain types of cancer.^2^ White AT is by far the largest storage site of lipids in the body in the form of neutral lipids, for example, triglycerides (TG) and cholesterol-esters. Lipids are deposited by adipocytes within lipid droplets (LDs) and can be released on demand, in the form of free fatty acids (FFAs), by associated lipases and taken up by other tissue for *β*-oxidation and subsequent ATP generation.^3, 4^

Nutrient restriction (NR) has been suggested to positively have an impact on human health and extend lifespan in several organisms, including *S. cerevisiae*, *C. elegans*, *D. melanogaster*, mouse and human.^5, 6^ NR undoubtedly represents the most efficient strategy reducing visceral AT, suggesting an inverse relationship between AT expansion and lifespan.^7^ Although it is not still entirely clear, NR is able to induce cellular responses culminating in increased stress resistance and longevity.^6^

The forkhead homeobox type O1 (FoxO1) transcription factor is a critical mediator of the cellular stress response and has been implicated in many nutrient-regulated processes.^8^ FoxO1 modulates lipid metabolism in AT through regulation of adipocyte size and the expression of AT-specific gene such as adipose triglyceride lipase (ATGL), the rate-limiting enzyme involved in the breakdown of TG stored into LDs.^9^

An alternative way to obtain FFAs from LDs has been firstly discovered in hepatocytes, which consists in LDs breakdown through autophagy by lysosomal lipases.^10^ This selective autophagy, named lipophagy, has been observed also in other cells including fibroblasts,^11^ neurons^12^ and even cancer cells,^13^ suggesting a generalized function of autophagy in cellular lipid mobilization. It has been demonstrated that intracellular lipid mobilization is particularly advantageous during NR, and lipophagy-mediated FFAs liberation essentially serves to maintain cellular energy homeostasis.^10, 14^

In AT, the role of autophagy is still controversial. Indeed, it regulates AT development, being essential for adipocytes differentiation.^15^ Accordingly, increased autophagy in AT has been associated with obesity and type-2 diabetes in mice and humans.^16, 17^ More recently, autophagy has been implicated in LDs degradation in fat cells both under basal and hormone-stimulated lipolysis,^18^ thus implicating it in FFAs release from AT and possibly fat mass decrease.

It is now clearly evident that an imbalance between the hydrolysis and synthesis of TG is involved in excessive fat pad accumulation and critical for the development of age-related metabolic disorders. For this reason, the manipulation of lipid metabolism at pharmacological level represents an attractive strategy to extend life and healthspan. Among the emerging antiaging drugs, metformin (Metf) is included.^19, 20, 21^ It is currently used as an oral antidiabetic particularly in overweight and obese subjects.^22^ Besides the well-recognized hypoglycemizing action, thanks to its ability to increase peripheral glucose uptake, Metf has been found to induce autophagy.^23, 24^ Metf was shown to cause a mild energetic drop thus mimicking a NR-related state.^19, 25^ Notwithstanding, the exact mechanisms behind the geroprotective effect of Metf are not still clearly established, particularly those related to regulation of lipid metabolism in AT.

Despite adipocytes are considered the main cells able to accumulate lipids in the form of TG, scarce evidence exists regarding the role of autophagy in regulation of TG breakdown. In this work, we have investigated the role of FoxO1 in modulating lysosomal lipid catabolism during NR in adipocytes and tested the potential use of Metf as a pro-lypolytic drug via the induction of FoxO1-mediated lipophagy in AT.

## Results

### FoxO1 modulates Lipa expression upon nutrient restriction and Metf treatment in adipocytes

**Figure 1:**
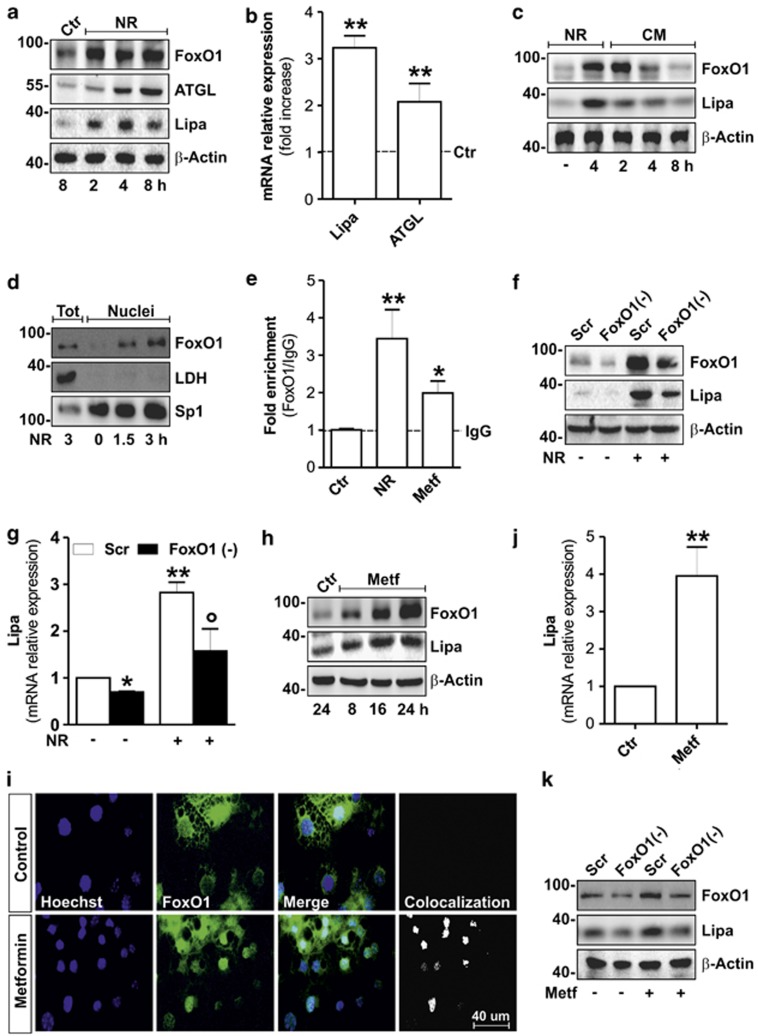
FoxO1-mediated lysosomal acid lipase (Lipa) induction in NR and Metf-treated 3T3-L1 adipocytes. (**a**) Western blot of FoxO1, ATGL and Lipa in total protein extracts from 3T3-L1 adipocytes at different times of NR. (**b**) RT-qPCR analysis of relative Lipa and ATGL mRNA levels in 3T3-L1 after 4 h from NR. Dashed line indicates the mRNA value of controls. (**c**) After 4 h from NR, 3T3-L1 adipocytes were refed with complete cell culture medium (CM) up to 8 h. Total protein extracts were used for western blotting analysis of FoxO1 and Lipa. (**d**) Western blot of FoxO1 in total and nuclear protein extracts from 3T3-L1 adipocytes at different times of NR. (**e**) ChIP assay was carried out on crosslinked nuclei from 3T3-L1 adipocytes subjected to NR for 4 h and Metf for 16 h by using FoxO1 antibody followed by qPCR analysis of FoxO1RE on *Lipa* promoter (*−*51 bp). Dashed line indicates the IgG value. (**f** and **g**) 3T3-L1 adipocytes were transfected with siRNA against FoxO1 (FoxO1(−)) or with a scramble siRNA (Scr). Western blot of FoxO1 and Lipa (**f**) and RT-qPCR analysis of relative Lipa mRNA level (**g**) were performed in 3T3-L1 adipocytes 4 h after NR. (**h**) Western blot of FoxO1 and Lipa in 3T3-L1 adipocytes at different times of 5 mM Metformin (Metf) treatment. (**i**) Confocal analysis of FoxO1 localization in 3T3-L1 adipocytes treated with 5 mM Metf for 16 h. Nuclei were stained with Hoechst 33342. Colocalization plugin (ImageJ Software) was used to identify FoxO1-Hoechst colocalization (white spots). (**j**) RT-qPCR analysis of relative Lipa mRNA level were performed in 3T3-L1 adipocytes treated with Metf for 16 h. (**k**) 3T3-L1 adipocytes were transfected with siRNA against FoxO1 (FoxO1(−)) or with a scramble siRNA (Scr). Western blot of FoxO1 and Lipa was performed in 3T3-L1 adipocytes treated with 5 mM Metf for 24 h. All values are given as mean±S.D. (*n*=4). *P<0.05, **P<0.01 *versus* controls. **°***P*<0.05 *versus* NR

The nutrient-sensing FoxO1 transcription factor regulates ATGL expression promoting lipid catabolism in adipose cells.^9^ Interestingly, it has been recently reported that the FoxO1 homolog (dFOXO) induces lysosomal acid lipase (Lipa) in *D. melanogaster* participating in lipid catabolism during fasting.^26^ On the basis of this evidence, we asked whether NR could induce Lipa expression in mammalian adipocytes and FoxO1 could mediate this event. In 3T3-L1 murine adipocytes, we observed a progressive increase of FoxO1 protein level during NR (Figure 1a), which was accompanied by a time-dependent induction of Lipa and ATGL protein levels (Figure 1a). In particular, we detected an earlier induction of Lipa (as soon as 2 h) with respect to ATGL (starting at 4 h). Further, a concomitant increased mRNA expression of Lipa and ATGL was detected in 3T3-L1 adipocytes 4 h after NR (Figure 1b). The nutrient-sensing feature of FoxO1 and Lipa was confirmed by refeeding NR 3T3-L1 adipocytes with complete cell culture medium. Indeed, Figure 1c shows that FoxO1 protein returns to basal level as soon as 4 h from nutrients replenishment. Concomitantly, a reduction of Lipa protein levels was observed. Similar results were obtained by analyzing ATGL protein levels during refeeding of NR 3T3-L1 adipocytes (Supplementary Figure 1A).

Being the regulatory role of FoxO1 on ATGL induction already demonstrated in mammals,^9^ we focused our work on the control of FoxO1 on Lipa gene expression during NR. FoxO1 orchestrates the expression of its target genes primarily translocating into nuclear compartment under several stress stimuli.^27^ As expected, NR promoted a prompt time-dependent FoxO1 nuclear accumulation (Figure 1d). Successively, to determine whether Lipa was a direct target of FoxO1 activation, we analyzed its promoter and found one *TAAACT*-binding site (FoxO1RE) located at −51* *bp from the start codon. Chromatin immunoprecipitation coupled with quantitative PCR (ChIP-qPCR) carried out on NR 3T3-L1 adipocytes revealed about threefold increase of FoxO1 binding to FoxO1RE when compared with controls (Figure 1e). To confirm the orchestrating role of FoxO1 in Lipa expression, we downregulated FoxO1 by RNAi (FoxO1(−)) in NR 3T3-L1 adipocytes. Accordingly, FoxO1(−) cells displayed diminished levels of Lipa protein (Figure 1f and Supplementary Figure 1B) and mRNA (Figure 1g).

Some reports suggest that Metf can extend lifespan and ameliorate healthspan in mammals by inducing a NR-like state.^19^ A potential NR-mimicking effect of Metf has been related to its efficiency to reduce fat mass.^28, 29, 30^ However, even if the mechanisms by which NR reduces fat mass are widely documented, those regarding Metf remain unknown. In order to test whether Metf could affect lipid catabolism by the above-described pathway, we added this drug to 3T3-L1 adipocytes. Metf (5 mM) stimulated a time-dependent increase of Lipa, starting at 8 h of treatment (Figure 1h and Supplementary Figure 1C). This event was associated with FoxO1 upregulation (Figure 1h) and its nuclear translocation, as assessed by both confocal microscopy (Figure 1i) and western blot analysis on nuclear protein extracts (Supplementary Figure 1D). ChIP-qPCR analysis revealed that FoxO1-binding activity on Lipa promoter was significantly enhanced in 3T3-L1 adipocytes treated with Metf for 16 h (Figure 1e) and this event was associated with increased Lipa mRNA (Figure 1j). Moreover, similar to NR, Lipa upregulation was buffered in FoxO1(−) cells treated with Metf (Figure 1k), further corroborating the implication of FoxO1 in the modulation of Lipa expression.

**Figure 2:**
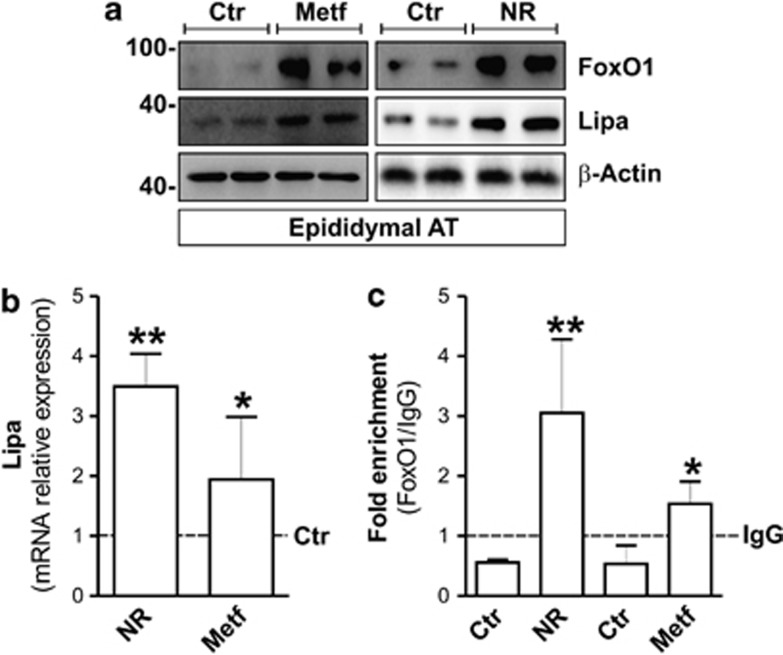
NR and Metf promote FoxO1-mediated Lipa upregulation in visceral AT of adult mice. (**a**) Adult C57/BL6 mice (5 months) were nutrient restricted (NR) by 24 h fasting or treated for 10 days with Metf (400 mg/kg) dissolved in drinking water (*n*=4 mice per group). Western blot of FoxO1 and Lipa in total protein extracts of explanted visceral (epididymal) AT. (**b**) RT-qPCR analysis of relative Lipa mRNA levels in NR- and Metf-treated visceral AT from two representative animals. (**c**) ChIP assay was carried out on crosslinked nuclei from NR- and Metf-treated visceral AT using FoxO1 antibody followed by qPCR analysis of FoxO1RE on *Lipa* promoter (*−*51 bp). Dashed line indicates the IgG value. *β*-actin was used as loading controls. All values are given as mean±S.D. *P<0.05, **P<0.01 *versus* controls

We thus attempted at comparing the effect of NR and Metf *in vivo*. To this end, adult mice (5 months) were nutrient restricted (NR) by 24 h fasting or treated with 400 mg/kg of Metf for 10 days. Figure 2a shows that visceral (epididymal) AT of Metf-treated mice displays an increased FoxO1 protein level that was similar to that observed in mice subjected to NR. Coherently, upon Metf treatment heightened Lipa upregulation was also observed both in terms of protein (Figure 2a) and mRNA (Figure 2b). Moreover, an increased FoxO1 binding on Lipa promoter was effective both in NR- and Metf-treated mice (Figure 2c), involving FoxO1 in modulation of Lipa also in *in vivo*.

### Metabolic stress induces lipophagy in adipocytes

**Figure 3:**
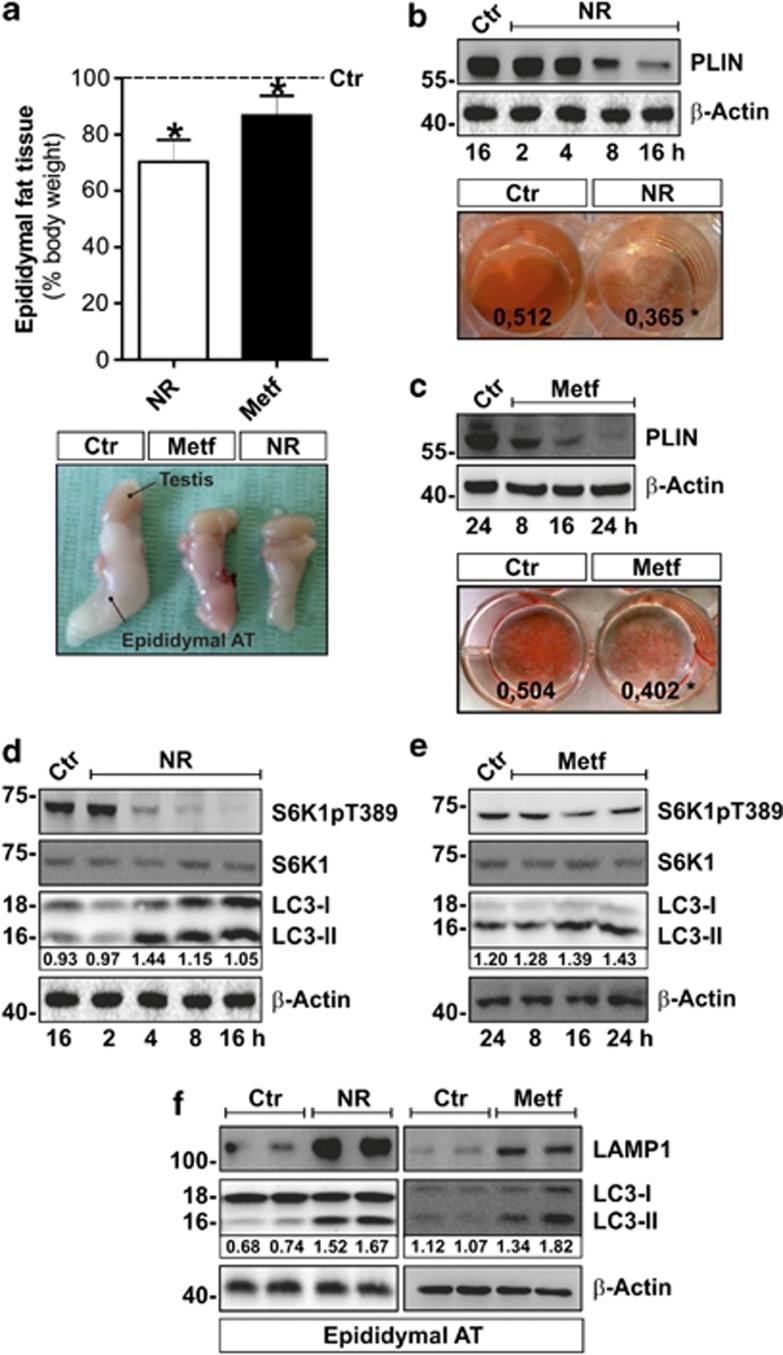
Metabolic stress induces lipid catabolism and autophagy in adipocytes. (**a**) Upper panel: weights of visceral AT of mice subjected to NR or Metf treatment were expressed as percentage of body weight and compared with controls (dashed line). Bottom panel: representative photograph relative to visceral (epididymal) AT after NR or Metf treatments (*n*=4 mice per group). (**b**) Upper panel: western blot of PLIN in total protein extracts of 3T3-L1 adipocytes at different times of NR. Bottom panel: ORO staining of 3T3-L1 adipocytes after 6 h of NR. Eluted ORO absorbance is numerically reported. (**c**) Upper panel: western blot of PLIN in total protein extracts of 3T3-L1 adipocytes at different times of Metf treatment. Bottom panel: ORO staining of 3T3-L1 adipocytes after 6 h of NR. Eluted ORO absorbance is numerically reported. (**d** and **e**) Western blot of phosphoactive (S6K1pT389) and basal forms of S6K1, LC3-I and LC3-II in total protein extracts of 3T3-L1 adipocytes at different times of NR (**d**) or Metf treatment (**e**). Values of LC3-II/LC3-I ratio were reported as relative density of immunoreactive bands (**f**) Western blot of LAMP1 and LC3 in visceral AT of NR or Metf-treated mice (*n*=4 mice per group). Values of LC3-II/LC3-I ratio were reported as relative density of immunoreactive bands. *β*-actin was used as loading control. All values are given as mean±S.D. *P<0.05 *versus* controls. *In vitro* data are representative of at least three independent experiments

Although we did not reveal any changes in total body weight of NR- and Metf-treated mice, AT mass underwent a significant reduction (Figure 3a). NR and Metf were effective also in reducing intracellular TG content in 3T3-L1 adipocytes. In particular, by using Oil Red-O (ORO) staining, we found a significant decrease of stored TG both during NR (Figure 3b) and Metf treatment (Figure 3c). Accordingly, perilipin (PLIN), a protein specific for the LDs surface, progressively declined in 3T3-L1 adipocytes during such treatments (Figures 3b and c).

These results, together with the outlined Lipa induction, prompted us to evaluate whether autophagy was involved in lipid degradation. Thus, canonical autophagic markers were examined during either NR or Metf treatment in adipose cells. Although at different times and with dissimilar efficiency, we found that the lipidated form of LC3 (LC3-II) as well as LC3-II/LC3-I ratio resulted progressively increased in 3T3-L1 adipocytes either subjected to NR (Figure 3d) or treated with Metf (Figure 3e). The same results were obtained in epididymal AT of NR- and Metf-treated mice (Figure 3f). Successively, we quantified the level of autophagy through cytofluorimetric analysis by staining cells with acridine orange, a lysotropic dye accumulating in acidic organelles.^31^ Interestingly, either NR or Metf were able to increase the rate of adipocytes that underwent autophagy (Supplementary Figure 2A). Finally, during NR and Metf treatment we observed a reduction of phosphoactive form of p70 S6 kinase (S6K1; Figures 3d and e), a well-known downstream target of the antiautophagic mTOR.^32^

To understand the contribution of autolysosomal activity, we analyzed the content of lysosome-associated membrane protein 1 (LAMP1), a component of the lysosomal membrane. In line with the results showing the accumulation of lysosomal-resident Lipa, NR and Metf treatment upregulated both protein (Figure 3f) and mRNA (Supplementary Figure 2B) levels of LAMP1 in AT.

**Figure 4:**
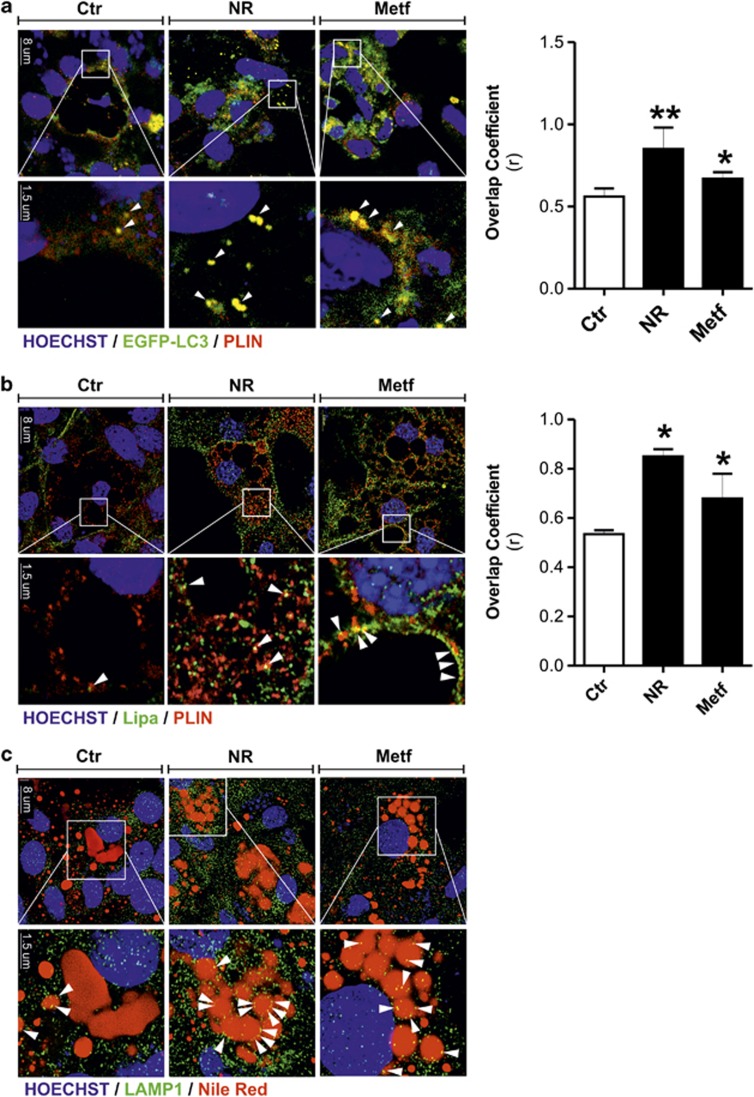
Metabolic stress triggers lipophagy in adipocytes. (**a**) 3T3-L1 adipocytes were transfected with EGFP-LC3 expression vector (green) and subjected to NR or treated with Metf. Cells were immunostained with PLIN antibody (red). (**b**) 3T3-L1 adipocytes were subjected to NR or treated with Metf for 8 h. Cells were immunostained with Lipa (green) and PLIN (red) antibodies. (**c**) 3T3-L1 adipocytes were subjected to NR or treated with Metf for 8 h. Cells were immunostained with LAMP1 antibody (green). LDs were visualized by using Nile Red (red). Nuclei were counterstained with Hoechst 33342 (blue). Arrowheads indicate examples of colocalization points. Overlap coefficients were calculated by JACoP (plugin of ImageJ Software) in at least four different images. All values are given as mean±S.D. *P<0.05, **P<0.01 *versus* controls

To confirm the involvement of autophagy in lipid catabolism, we carried out colocalization analyses by confocal microscopy. 3T3-L1 adipocytes were transfected with green fluorescent protein-tagged LC3 expression vector (enhanced green fluorescent protein (EGFP)-LC3) and stained with PLIN to locate the autophagolysosome-targeted LDs. Under basal conditions, EGFP-LC3 signal appeared substantially diffused, indicating a low rate of autophagy; however, a small amount of EGFP-LC3 colocalized with PLIN (Figure 4a). Upon 16 h of NR or Metf treatment, there was a marked increase of punctate EGFP-LC3 that tightly colocalized with PLIN (Figure 4a).

Next, we examined the possible Lipa association with LDs surface marked with PLIN. Under resting condition, a minor subset of Lipa was found to colocalize with PLIN (Figure 4b). Upon 8 h of NR and Metf treatment, there was an enhancement of Lipa-derived signal and its redistribution around LDs (Figure 4b). Moreover, a significant increased colocalization of LIPA with PLIN was observed in NR- and Metf-treated cells with respect to control (Figure 4b). Successively, to further confirm the effectiveness of NR and Metf treatment on packaging and delivery of lysosomes to LDs, we probed LDs by Nile Red and examined the distribution of lysosomes by LAMP1 staining. According to the above-described results, an enhanced LAMP1 redistribution around LDs was observed in 3T3-L1 adipocytes after NR and Metf treatment (Figure 4c), thus finally implying lipophagy in adipocyte lipid catabolism.

### AMPK restrains energetic catastrophe driving Lipa-released fatty acids to oxidation

**Figure 5:**
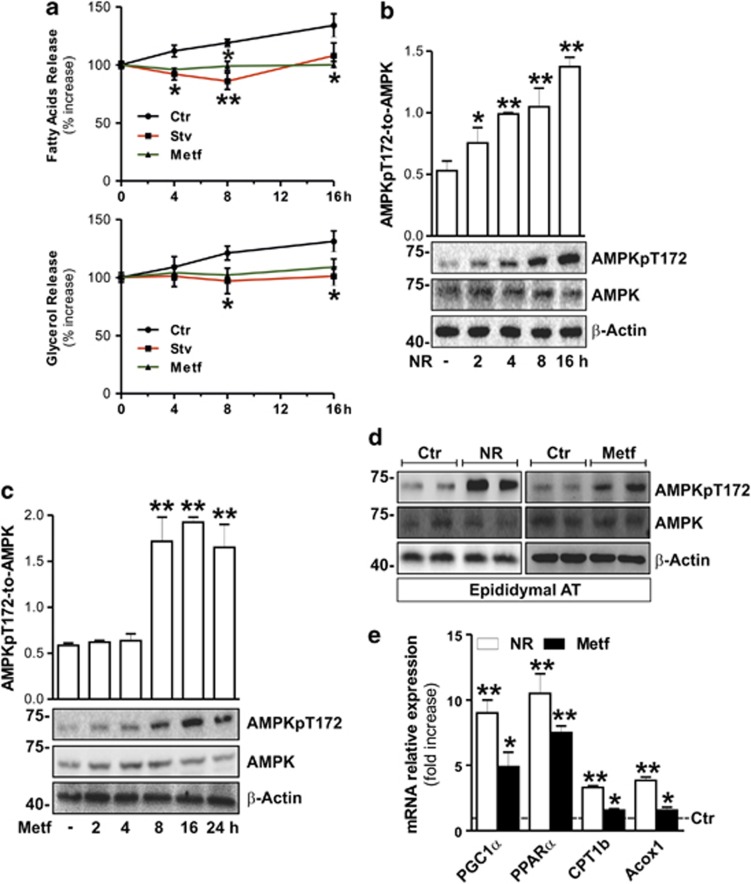
NR and Metf activate AMPK and lipid oxidative genes in adipocytes. (**a**) FFAs (upper panel) and glycerol (bottom panel) were enzymatically detected in culture medium at different times of NR or Metf treatment in 3T3-L1 adipocytes. All values are expressed as percentage of increase with respect to time 0. (**b** and **c**) Western blot of phosphoactive (AMPKpT172) and basal forms of AMPK in total protein extracts from 3T3-L1 adipocytes at different times of NR (**b**) or Metf (**c**) treatment. Relative density of immunoreactive bands was reported as AMPKpT172/AMPK (upper panels). (**d**) Western blot of phosphoactive and basal forms of AMPK in total protein extracts from visceral (epididymal) AT of NR or Metf-treated mice. (**e**) RT-qPCR analysis of relative peroxisome proliferator-activated receptor gamma-1*α*, peroxisome proliferator-activated receptor-*α*, carnitine palmitoyltransferase 1b and acyl-CoA oxidase 1 mRNA levels were performed in visceral AT of NR- or Metf-treated mice. Dashed line indicates the mRNA value of controls (*n*=4 mice per group). *β*-actin was used as a loading control. All values are given as mean±S.D. *P<0.05, **P<0.01 *versus* controls. *In vitro* data are representative of at least three independent experiments

Interestingly, although we revealed a reduced TG content, no increase in glycerol and FFAs in culture medium of NR- and Metf-treated adipocytes were observed (Figure 5a). In particular, a reduced level of FFAs was detected in culture medium at earlier times of NR (Figure 5a: upper panel), implying that adipocytes preferentially use FFAs as an energetic reservoir during metabolic stress. These phenomena suggested that LDs-deriving FFAs might be funneled toward oxidation. It is well recognized that NR and Metf represent strong inducers of AMP-activated protein kinase (AMPK).^25, 33, 34, 35^ Generally, during metabolic stress AMPK assures cell survival maintaining adequate cellular energy balance by modulating the expression of genes involved in ATP-generating pathways through FFAs oxidation.^36, 37^ On the basis of these findings, we firstly verified whether the energy-sensing AMPK could be modulated by NR and Metf treatment in adipocytes. We found that, after such treatments, a time-dependent increase of the phosphoactive form of AMPK (AMPKpT172) was triggered in 3T3-L1 adipocytes (Figures 5b and c). Similarly, AT from NR- and Metf-treated mice showed a phosphoactivation of AMPK (Figure 5d). AMPK activation was also accompanied by an increased expression of key downstream genes controlling lipid oxidation, that is, peroxisome proliferator-activated receptor gamma-1*α*, peroxisome proliferator-activated receptor-*α*, carnitine palmitoyltransferase 1b and acyl-CoA oxidase 1 (Figure 5e).

**Figure 6:**
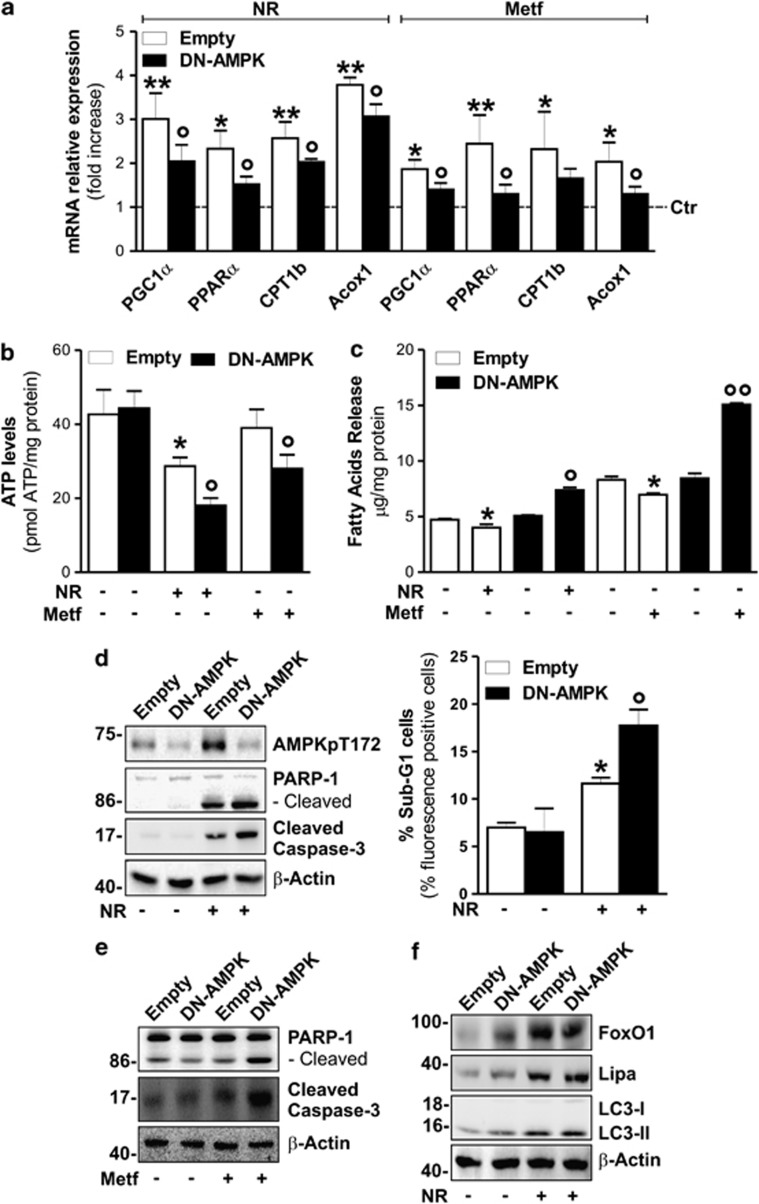
AMPK drives Lipa-released FFAs oxidation restraining energetic catastrophe. (**a**) 3T3-L1 cells were transfected with DN-AMPK or empty vector. RT-qPCR analysis of relative peroxisome proliferator-activated receptor gamma-1*α*, peroxisome proliferator-activated receptor-*α*, carnitine palmitoyltransferase 1b and acyl-CoA oxidase 1 mRNA levels were performed after 4 h of NR or 16 h of Metf treatment. Dashed line indicates the mRNA value of untreated DN-AMPK cells (Ctr). mRNA levels of untreated cells transfected with empty vector were similar to untreated DN-AMPK cells (data not shown). (**b**) Cheminoluminescent assay of ATP level in 3T3-L1 adipocytes transfected with DN-AMPK or empty vector after 8 h NR or 16 h Metf treatment. ATP level was expressed as pmol ATP per mg protein. (**c**) After 8 h of NR or 16 h Metf treatment, FFAs were enzymatically detected in culture medium of 3T3-L1 adipocytes transfected with DN-AMPK or empty vector. Values were expressed as *μ*g FFAs per mg protein. (**d**) Left panel: western blot of AMPKpT172, PARP-1 and cleaved form of caspase-3 in 3T3-L1 adipocytes transfected with DN-AMPK or empty vector and subjected to 8 h NR. Right panel: cytofluorimetric analysis of apoptosis in DN-AMPK cells subjected to 8 h NR. (**e**) Western blot of PARP-1 and cleaved form of caspase-3 in 3T3-L1 adipocytes transfected with DN-AMPK or empty vector and treated with Metf for 16 h. (**f**) Western blot of FoxO1, Lipa, LC3 in 3T3-L1 adipocytes transfected with DN-AMPK or empty vector and subjected to 4 h NR. *β*-actin was used as loading control. All values are given as mean±S.D. *P<0.05, **P<0.01 *versus* controls; **°***P*<0.05, **°°***P*<0.01 *versus* Metf treatment. All data are representative of at least three independent experiments

Similar to in *in vivo* data, we found that also 4 h NR and 16 h Metf treatment elicited a prominent increase of lipid oxidative genes (Figure 6a). To imply AMPK in the adaptive response to NR and Metf, we transfected 3T3-L1 adipocytes with a dominant-negative form of AMPK (DN-AMPK). DN-AMPK cells showed a dampened expression of lipid oxidative genes upon NR and Metf treatments (Figure 6a), which was accompanied by an energetic drop, as demonstrated by the decline of ATP levels (Figure 6b). Further, a massive release of FFAs in culture medium of DN-AMPK cells was revealed upon both NR and Metf treatment (Figure 6c), suggesting that, under this condition, liberated FFAs were not directed toward oxidation. Similar results were obtained by supplementing NR- and Metf-treated 3T3-L1 adipocytes with 20 *μ*M compound-C, a chemical inhibitor of AMPK (data not shown). Successively, we observed that upon NR, the inhibition of AMPK led to an exacerbated induction of apoptosis, as demonstrated by the enhanced levels of cleaved PARP-1 and caspase-3 (Figure 6d: left panel) as well as an augmented percentage of sub G1 cells (Figure 6d: right panel). DN-AMPK adipocytes showed increased susceptibility also to Metf; indeed, they displayed a higher degree of PARP-1 and caspase-3 cleavage at 16 h after Metf treatment (Figure 6e). Importantly, inhibition of AMPK activity in 3T3-L1 adipocytes did not significantly affect FoxO1-Lipa axis and LC3-II levels in 3T3-L1 adipocytes upon NR (Figure 6f), indicating that AMPK was not involved in orchestrating lipophagy.

**Figure 7:**
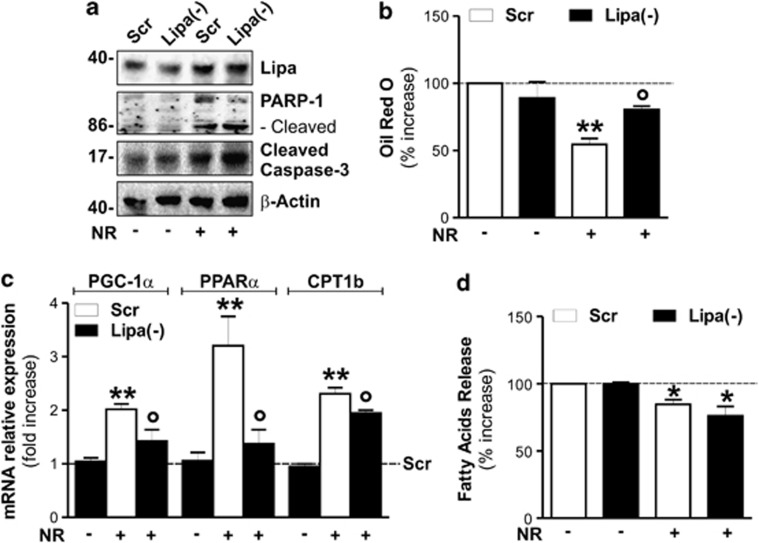
Lipa downregulation impairs lipid breakdown and elicits cell death in nutrient restricted adipocytes. (**a**) 3T3-L1 adipocytes were transfected with siRNA against Lipa (Lipa(−)) or with a scramble siRNA (Scr). Western blot of Lipa, PARP-1 and cleaved form of caspase-3 in total protein extracts from 3T3-L1 adipocytes after 4 h of NR. (**b**) TG content was quantified by ORO staining in fixed 3T3-L1 adipocytes 6 h after NR. (**c**) RT-qPCR analysis of relative peroxisome proliferator-activated receptor gamma-1*α*, peroxisome proliferator-activated receptor-*α* and carnitine palmitoyltransferase 1b mRNA levels was performed in 3T3-L1 adipocytes 4 h after NR. (**d**) FFAs were analyzed in culture medium 6 h after NR. β-actin was used as loading control. All values are given as mean±S.D. *P<0.05, **P<0.01 *versus* controls; **°***P*<0.05 *versus* NR treatment. All data are representative of at least three independent experiments

Finally, to better understand the role of Lipa upregulation in releasing FFAs under NR, we downregulated Lipa by RNAi (Lipa(−)) in 3T3-L1 adipocytes. As shown in Figure 7a, Lipa(−) cells were highly susceptible to NR, showing an increased rate of apoptosis, as assessed by the analysis of PARP-1 and caspase-3 cleavage. These events were associated with a significant reduction of the NR-mediated TG degradation (Figure 7b) and induction of lipid oxidative genes (Figure 7c). As expected, no changes were observed in FFAs extracellular release after Lipa downregulation (Figure 7d).

## Discussion

To date, FFAs release from adipocytes lipid stores has been ascribed to the activation of the cytosolic neutral lipases cascade, among which ATGL represents the rate-limiting enzyme. More recently, FFAs have been discovered to be liberated through an autophagy-mediated lipolysis, also termed lipophagy. Notwithstanding, the role of lipophagy in LDs remodeling in adipocytes has been poorly characterized. In this work, we have demonstrated that lipophagy represents an alternative pathway of TG degradation upon NR in adipocytes. Our findings are in line with the proposed implication of Lipa in mediating the mobilization of TG through lipophagy.^10^ In particular, by downregulating Lipa, we have shown that the prompt Lipa-mediated liberation of FFAs is mandatory to sustain energy production upon nutrient stress.

The nutrient-sensing FoxO1 transcription factor is currently being suggested to improve lipid catabolism during NR by managing the expression of ATGL in murine adipocytes^38^ and lysosomal lipase in *D. melanogaster*.^26^ Herein we have given further efforts regarding the contribution of FoxO1 in the control of lipid catabolism in mammalian adipocytes, identifying also Lipa as FoxO1 gene target upon NR. In particular, we outlined that NR promotes FoxO1 nuclear accumulation and this is mandatory for Lipa gene transcription in adipocytes. Our data suggest that FoxO1 activation provides an additional pathway to consume stored TG in AT independently of hormonal-mediated canonical lipolysis, supporting the notion that the lack of FoxO1 leads to expanded adipose mass and consequently reduced lifespan in mice.^39, 40^ Interestingly, FoxO1 is a master regulator of the expression of other autophagic genes;^8^ thus, it can be postulated that also in our experimental model, apart from inducing Lipa-mediated lipolysis, FoxO1 can modulate the constitution of the overall autophagy machinery.

One of the goals of recent aging research is the identification of drugs that lower the incidence of age-related disorders by promoting youthful physiology. A candidate chemical for achieving this extended healthspan is Metf, a biguanides widely used to treat type-2 diabetes and linked to promoting a broad range of health benefits.^19, 22^ Metf has recently been reported to have a broad range of beneficial effects on visceral AT metabolism.^41^ Until now, the molecular mechanisms by which Metf reduces fat mass are unclear. Interestingly, we found that Metf-treated adipose cells show a NR-like transcriptional profile, specifically characterized by FoxO1-mediated Lipa upregulation and enhanced expression of lipid oxidative genes. Further, similar to NR, Metf triggers a lysosomal-mediated lipolysis leading to TG degradation.

In our work, we have also underlined the overlapping effects of Metf and NR in adipocytes pointing out that they both activate AMPK. In particular, we clarified that, similar to NR, Metf activates AMPK-mediated FFAs oxidation, limiting their extracellular release from adipose cells.^42, 43, 44^ Our data reinforce the evidence of the lowering effects of Metf on plasma FFAs, which are notably increased during age-related pathological conditions^45, 46^ and unveil a mechanism of FFAs oxidation in adipose cells that likely limits the excessive FFAs release during NR.

**Figure 8:**
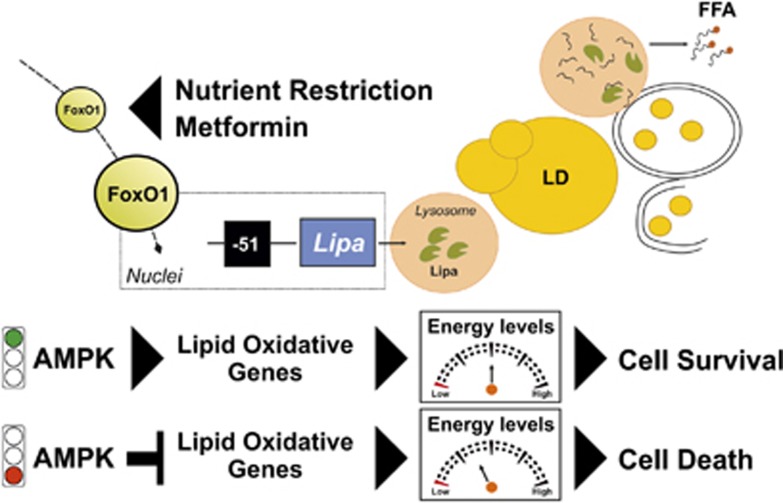
Schematic diagram of the molecular pathways activated in adipocytes upon metabolic stress. NR or Metf endorse similar stress resistance responses in adipocytes. FoxO1 delocalizes into nuclear compartment and this event is crucial to upregulate Lipa, which is mandatory for lipophagic induction. Lipophagy promotes fatty-acid release, which are directed toward oxidation by AMPK. These events confer cell survival in metabolically stressed adipocytes. FoxO1, forkhead homeobox type protein O1; Lipa, lysosomal acid lipase; LD, lipid droplet; FFA, free fatty acids

In summary, FoxO1 represents a master regulator both of canonical and lysosomal-mediated lipid catabolism in adipocytes under metabolic stress. Further, during NR an immediate adaptive lipid catabolic process in adipocytes is activated that is favored by a prompt Lipa upregulation that precedes cytoplasmic ATGL induction. Lipa upregulation represents a resourceful response that promotes FFAs release necessary to maintain ATP levels in metabolically stressed fat cells. In this scenario, we have evidenced that AMPK is the ‘stationmaster' in adipose lipid metabolism, driving Lipa-released FFAs toward oxidation, thus providing stress resistance (Figure 8). Finally, our findings give further effort to the evidence that Metf has a significant NR-mimicking potential in adipocytes, suggesting its appetizing employment in the onset of aging where an increase of visceral AT and metabolic disorders occur.

## Materials and Methods

### Mice and treatments

We conducted all mouse experimentations in accordance with accepted standard of humane animal care and with the approval by relevant national (Ministry of Welfare) and local (Institutional Animal Care and Use Committee, Tor Vergata University) committees. C57BL/6 adult (5 months) male mice were purchased from Harlan Laboratories S.r.l. (Urbino, Italy).

For NR *in vivo* experiment, eight mice were equally and randomly divided into two groups: *ad libitum* fed (Ctr) and nutrient restricted (NR). NR was performed by 24 h fasting. In this period, each NR mouse had free access to water.

For *in vivo* Metf treatment, eight mice were equally and randomly divided into two groups: untreated (Ctr) and Metf-treated group (Metf). Metf was orally supplied in drinking water (400 mg/kg) for 10 days.

After cervical dislocation, epididymal AT was explanted and immediately frozen on dry ice and stored at −80 °C.

### Cell lines, treatments and transfections

3T3-L1 murine pre-adipocytes were purchased from ATCC (American Type Culture Collection, Bethesda, MD, USA) and grown in DMEM supplemented with 10% new born serum, 1% pen/strep mix and 2 mM glutamine (Lonza Sales, Basel, Switzerland) and cultured as previously described.^47^ 3T3-L1 cells were differentiated in adipocytes as reported by Chakrabarti and Kandror^9^ and all experiments were performed in fully differentiated adipocytes (day 8). NR experiments were carried out by using DPBS with calcium and magnesium and supplemented with 1% pen/strep mix (Lonza). Metformin (Sigma-Aldrich, St. Louis, MO, USA) was dissolved in PBS and added in serum-free culture medium at a final concentration of 5 mM. AMPK inhibitor compound C (Sigma-Aldrich) was solubilized in DMSO and added in culture medium 1 h before NR or Metf treatment at a final concentration of 20 *μ*M and maintained throughout the experiment.

Fully differentiated adipocytes were transfected with FoxO1, Lipa or scramble siRNAs (Santa Cruz Biotechnology, Dallas, TX, USA) by using DeliverX Plus kit (Affymetrix, Santa Clara, CA, USA). Alternatively, they were transfected with Pc-DNA3.1 plasmid (Life Technologies, Monza, Italy) containing EGFP-LC3 or DN-AMPK cDNA by using Turbofect Transfection Reagent (Thermo Scientific, Waltham, MA, USA). Adipocytes were subjected to NR or treated with Metf 48 h after transfection.

### Gel electrophoresis and western blotting

Cells and AT were lysed in RIPA buffer (50 mM Tris-HCl pH 8.0, 150 mM NaCl, 0.1% SDS, 0.5% sodium deoxycholate and 1% NP-40) supplemented with protease inhibitors cocktail (Merck Millipore, Darmstadt, Germany). Western blotting analysis was performed as previously described^48, 49^ by using the following polyclonal antibodies: ATGL, *β*-Actin, LDH, Sp1 and PLIN1, AMPK (Santa Cruz Biotechnologies), Lipa (Novus Biologicals, Littleton, CO, USA), LC3 (Sigma-Aldrich), LAMP1, S6K1 (Abcam, Cambridge, UK) and cleaved caspase-3, FoxO1, PARP-1, S6K1pT389, AMPKpT172 (Cell Signalling Technologies, Danvers, MA, USA). Immunoblots reported in the figures are from one experiment representative of four that gave similar results (*in vitro* experiments). For *in vivo* experiments, immunoblots of two representative animals out of four (for each group) were reported.

### RT-qPCR analysis

RT-qPCR analysis was carried out as previously described.^48^ Briefly, total RNA was extracted using TRI reagent (Sigma-Aldrich). Three micrograms of RNA was used for retrotranscription with M-MLV (Promega, Madison, WI, USA). qPCR was performed in triplicates by using validated qPCR primers (BLAST), Ex TAq qPCR Premix (Lonza Sales) and the Real Time PCR LightCycler II (Roche Diagnostics, Indianapolis, IN, USA). mRNA levels were normalized to *β*-actin mRNA, and the relative mRNA levels were determined by using the 2^−ΔΔ*C*t^ method.

### Preparation of cytoplasmic and nuclear extracts

Cell pellets were resuspended in lysis buffer containing 10 mM NaCl, 3 mM MgCl_2_, 10 mM Tris-HCl, pH 7.8, 0.5% NP-40, 1 mM DTT and protease inhibitors. Nuclei were collected by centrifugation at 2000 × *g* for 5 min at 4 °C. Supernatant (cytoplasmic fraction) was collected and pellet (nuclei) was resuspended in 50 *μ*l of HSB buffer (50 mM Tris-HCl, pH 7.5, 400 mM NaCl, 1 mM EDTA, 1 mM EGTA, 1% Triton X-100, 0.5% NP-40, 10% glycerol and protease inhibitors) and incubated 30 min on a rotating wheel at 4 °C. Extracts were centrifuged at 22000 × *g* to remove nuclear debris and the supernatants (nuclear proteins) were used for western blot, oligonucleotide pull-down and ChIP assays.

### Chromatin immunoprecipitation assay

ChIP assay was carried out as previously described.^48^ Briefly, after crosslinking, nuclei extracted from 3T3-L1 adipocytes and visceral AT were fragmented by ultrasonication using 4 × 15 pulse (output 10%, duty 30%). Samples were precleared with preadsorbed salmon sperm Protein G agarose beads (1 h, 4 °C), and then overnight immunoprecipitation using anti-FoxO1 or control IgG antibody was carried out. After de-crosslinking (1% SDS at 65 °C for 3 h), qPCR was used to quantify the promoter binding with 30 cycles total (95 °C, 1 s; 60 °C, 30 s; 72 °C, 60 s). Results are expressed as fold enrichment with respect to IgG control.

### Confocal microscopy

Cells were seeded directly on glass coverslips, fixed with 4% paraformaldehyde and permeabilized by incubation with 0.2% Triton X-100. 3T3-L1 adipocytes were incubated with anti-FoxO1, anti-PLIN (Cell Signalling Technologies), anti-LAMP-1 (Abcam) and anti-Lipa (Novus Biologicals). After staining with the appropriate AlexaFluor-conjugated secondary antibody (Life Technologies), confocal images were visualized with an Olympus Fluoview 1000 Confocal Laser Scanning System (Applied Precision Inc., Issaquah, WA, USA). Nuclei and LDs were stained with Hoechst 33342 (10 *μ*g/ml) and Nile Red (1 *μ*g/ml), respectively.

For nuclear FoxO1 localization, Colocalization plugin (ImageJ Software, Bethesda, MD, USA) was used. For detection of lipophagy, overlap coefficients (Lipa/PLIN, EGFP-LC3/PLIN) were calculated by using JACoP plugin (ImageJ Software). Lipa/PLIN colocalization was analyzed on 3T3-L1 cells subjected to NR and Metf treatment for 8 h, time when both proteins were still well detectable. EGFP-LC3/PLIN colocalization was analyzed at 16 h, time when LC-3II was significantly increased upon both NR and Metf treatment.

### TG staining, lipolysis assay and ATP

TG were visualized by ORO staining as previously described^47^ and quantification was performed by extraction with 4% IGEPAL in isopropanol followed by 550 nm absorbance analysis. FFAs were detected in culture medium by using FFAs quantification colorimetric kit (BioVision, Milpitas, CA, USA) according to the manufacturer's instructions. Alternatively, lipolysis was assayed by detecting glycerol content in culture medium by using the Free Glycerol Reagent (Sigma-Aldrich) according to the manufacturer's instructions.

ATP level was detected by using ATP Bioluminescence assay kit (Roche Diagnostics) on total cell extracts and values were normalized to protein content.

### Determination of apoptosis by cytofluorimetric analysis

Cells were stained with 50 *μ*g/ml propidium iodide (dissolved in 0.1% Triton X-100) and analyzed by a FACScalibur instrument (Beckton and Dickinson, San Jose, CA, USA). The percentage of apoptotic cells was evaluated according to Nicoletti *et al.*^50^ by calculating the peak area of hypodiploid nuclei (Sub G1).

Protein concentration was determined by the method of Lowry.

### Statistical analysis

The results are presented as means±S.D. Statistical evaluation was conducted by ANOVA, followed by the post Student–Newman–Keuls. Differences were considered to be significant at *P*<0.05.

## Abbreviations

ATGL: adipose triglyceride lipase
Lipa: lysosomal acid lipase
FoxO1: forkhead homeobox type protein O1
EGFP: enhanced green fluorescent protein
TG: triglycerides
FFAs: free fatty acids
NR: nutrient restriction
Metf: metformin
AT: adipose tissue
ORO: Oil Red-O
LDs: lipid droplets
