# Supplementary material for: FoxO1 controls lysosomal acid lipase in adipocytes: implication of lipophagy during nutrient restriction and metformin treatment
Source: Cell Death Dis. 2013 Oct 17;4(10):e861–. doi: 10.1038/cddis.2013.404 (PMC3920962; doi:10.1038/cddis.2013.404)
Supplement: Supplementary Information [file cddis2013404x1.pdf]

Inventory of Supplemental Information

Figure S1

Figure S2

Extended Experimental Procedures

Supplemental References

Figure S1

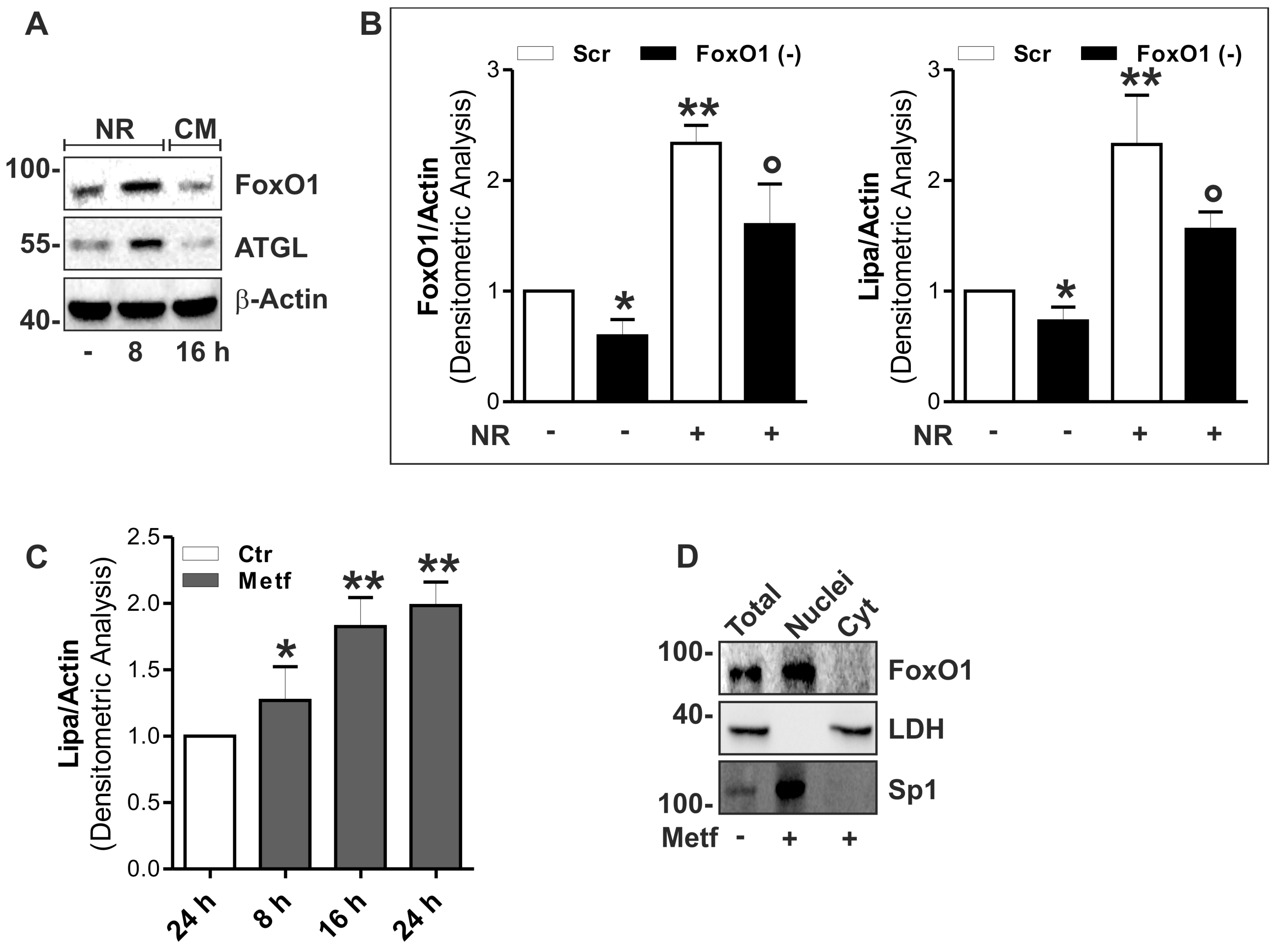

Figure S1. (**A**) After 8 h from NR, 3T3-L1 adipocytes were re-fed in complete cell culture medium (CM) up to 16 h. Total protein extracts were used for Western blot analysis of FoxO1 and ATGL. (**B**) 3T3-L1 adipocytes were transfected with siRNA against FoxO1 [FoxO1(-)] or with a scramble siRNA (Scr). Relative density of immunoreactive bands was reported as FoxO1/Actin (*left panel*) and Lipa/Actin (*right panel*) in 3T3-L1 adipocytes 4 h after NR. (**C**) Relative density of immunoreactive bands was reported as Lipa/Actin in 3T3-L1 adipocytes during 5 mM Metf treatment. (**D**) Western blot of FoxO1 in total, nuclear and cytoplasmic (Cyt) protein extracts from 3T3-L1 adipocytes 24 h after Metf treatment. β-Actin was used as loading control. All values are given as mean ± S.D. **P < 0.01 *vs* controls; ° P < 0.05 *vs* NR. All data are representative of at least 3 independent experiments.

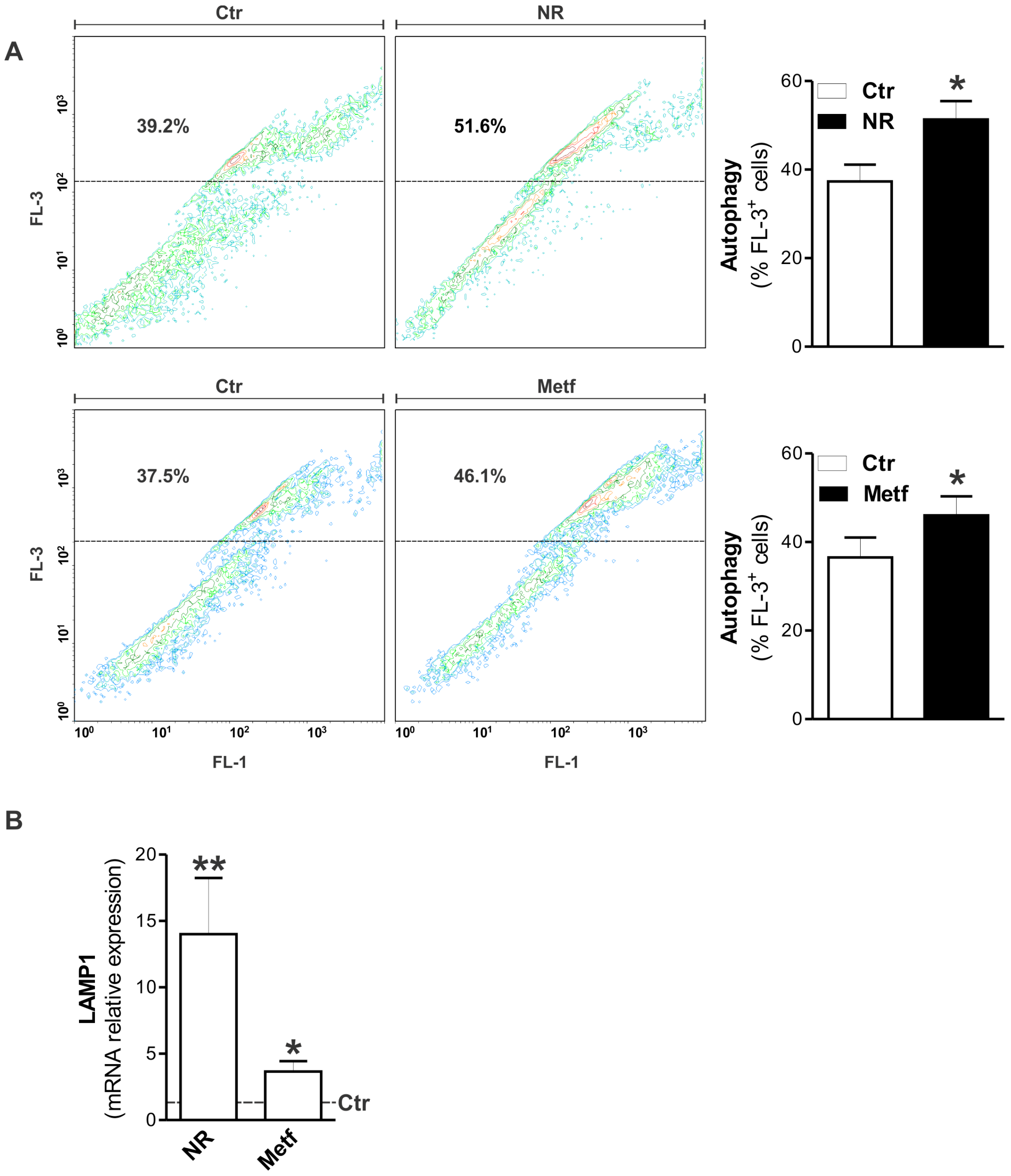

Figure S2. (**A**) Percentage of autophagic 3T3-L1 adipocytes 6 h after NR (*upper panel*) or 24 h after metf treatment (*lower panel*). (**B**) RT-qPCR analysis of relative LAMP1 mRNA levels in visceral AT of NR or Metf-treated mice (n = 4 mice/group). Dashed line indicates the mRNA value of controls. All values are given as mean ± S.D. *P < 0.05, **P < 0.01 *vs* controls. All in vitro data are representative of at least 3 independent experiments.

EXTENDEND EXPERIMENTAL PROCEDURES

Determination of autophagy by cytofluorimetric analysis

Autophagy was determined as previously described with some modifications [1]. Briefly, cells were stained with 500 nM acridine orange (Sigma Aldrich) for 15 min and analyzed by a FACScalibur instrument (Beckton and Dickinson, San Jose', CA, USA). The increase of FL-3 emitting cells (bright red) is considered proportional to the increase of intracellular acidification, which, in turn, mostly relies upon the accumulation of autolysosomes.

SUPPLEMENTAL REFERENCES

1. Desideri E, Filomeni G, and Ciriolo M.R. Glutathione participates in the modulation of starvation-induced autophagy in carcinoma cells. *Autophagy* 2012; **8**: 1769-1781.
